# Mortality and causes of deaths of inhabitants with renal dysfunction induced by cadmium exposure of the polluted Jinzu River basin, Toyama, Japan; a 26-year follow-up

**DOI:** 10.1186/1476-069X-13-18

**Published:** 2014-03-15

**Authors:** Shoko Maruzeni, Muneko Nishijo, Koshi Nakamura, Yuko Morikawa, Masaru Sakurai, Motoko Nakashima, Teruhiko Kido, Rie Okamoto, Kazuhiro Nogawa, Yasushi Suwazono, Hideaki Nakagawa

**Affiliations:** 1 Department of Public Health, Kanazawa Medical University, Uchinada 920-0293, Japan; 2 School of Nursing, Kanazawa Medical University, Uchinada 920-0293, Japan; 3 Department of Community Health Nursing, School of Health Sciences, Kanazawa University, Kanazawa 920-0942, Japan; 4 Department of Occupational and Environmental Medicine, Graduate School of Medicine, Chiba University, Chiba 260-8670, Japan

**Keywords:** Cadmium, Renal toxicity, A follow-up study, Mortality risk, Cancer, Ischemic heart diseases

## Abstract

**Background:**

We aimed to investigate the mortality and causes of deaths of inhabitants with renal dysfunction induced by cadmium (Cd) exposure caused by heavy environmental contamination.

**Methods:**

We conducted a 26-year follow-up survey targeting 7529 inhabitants of the Cd-polluted Jinzu River basin and 2149 controls from non-polluted areas who participated in urinary examinations for proteinuria and glucosuria conducted in 1979 to 1984. When the residents were divided into 4 groups, no finding group, glucosuria group, proteinuria group, glucoproteinuria group, mortality risk ratios for all and specific causes of these groups in the polluted area were compared with that of controls without glucosuria and/or proteinuria after adjustments for age at baseline, smoking status, and history of hypertension using Cox’s proportional hazard model.

**Results:**

The mortality risk ratios for all causes of proteinuria and glucoproteinuria in men and glucosuria, proteinuria, and glucoproteinuria in women of the polluted areas significantly increased compared with those of the controls with no urinary findings. Respiratory, renal, and cardiovascular diseases and diabetes in men, and all diseases except cerebrovascular diseases in women contributed toward an increased mortality of exposed glucoproteinuria groups, which involved chronic Cd toxicosis with renal tubular dysfunction. In women, the mortality risks for cancer of the colon and rectum, uterus and kidney and urinary tract were significantly higher in the exposed proteinuria and glucoproteinuria groups, suggesting associations between renal damage and cancer risk. In exposed women, the no finding group and glucoproteinuria group also showed increased mortality from ischemic heart diseases, indicating that all exposed women may be at risk for ischemic heart diseases. Although the control glucosuria and/or proteinuria group also showed high mortality for diabetes and renal diseases, the increased risk ratio for renal disease mortality was much higher in exposed subjects with urinary findings, particularly in women.

**Conclusions:**

These findings indicate that inhabitants with renal effects caused by Cd exposure had a poor life prognosis over long-term observation in both genders. Particularly in women, renal tubular dysfunction indicated by glucoproteinuria may increase mortality from cancer, ischemic heart diseases, and renal diseases.

## Introduction

Causes of deaths, particularly cancer mortality in Cd-exposed areas are an interesting issue. Cd and its compounds were classified as a human carcinogen by the International Agency for Research on Cancer [1,2]. Verougstraete et al. reviewed previous studies and reported that longitudinal studies have consistently shown that workers exposed to Cd are at a higher risk of lung cancer [3]. In a Belgian cohort study, Nawrot et al. reported a significant association between lung cancer risk and Cd exposure from aspiration of house dust containing Cd caused by past smelter emissions [4].

However, Sorahan et al. investigated mortality for lung cancer in factory workers exposed to Cd in the United Kingdom in a follow-up study from 1947 to 2000, but they detected neither significant increase in the mortality, nor a positive relationship between Cd exposure and lung cancer risk [5]. Moreover, in Japanese cohort studies in the Kakehashi River basin of Ishikawa prefecture, increased cancer mortality rate has not been reported except for an increased risk of cancer in women with renal tubular dysfunction during the early observation period of Cd-exposed inhabitants [6].

The Jinzu River basin in Toyama is the largest and highest Cd-polluted area in Japan and endemic for itai-itai disease, which is clinically characterized by renal tubular dysfunction caused by an advanced decrease in renal tubuli [7] and bone pain due to osteomalacia with Looser zones in roentgenograph of bone [8]. From 1979 to 1984, the Ministry of Environment in Japan conducted health-screening surveys targeting 7348 inhabitants in this polluted area and 2098 control individuals from several other areas in Toyama [9]. Based on this health survey, we followed up with these participants in the Jinzu River basin and control areas for 26 years and reported increased mortality risks for colorectal cancer, ischemic heart diseases, and renal diseases in all women of the Cd-polluted area compared with those in the control area. However, associations between mortality and renal tubular dysfunction have not been investigated in this area. Therefore, in the present study, we analyzed differences in mortality and causes of deaths in exposed residents with different urinary findings in the 1979-84 health survey indicating renal tubular dysfunction from those in the control area after adjusting for age, smoking status, and hypertension history, which are general risk factors for cancer and circulatory diseases.

## Methods

### Study subjects

A total of 7348 participants from a health-screening survey conducted in 1979–1984 (men, 3363; women, 3985), which included 97.6% of all inhabitants aged ≥ 50 years, were targeted in the present follow-up survey. Subjects lived in the Cd-polluted Jinzu River basin that included one part of Toyama city, all of Fuchu town, all of Ohsawano town, and one part of Yatsuo town. An additional 2098 inhabitants aged >50 years (men, 926; women, 1172) who participated in the health-screening survey (97.6% participation rate) from non-polluted areas in two towns and five cities were evaluated as controls in the present survey.

In the health-screening survey in 1979–1984, urinary quantitative protein measurement and glucose qualitative tests were performed for all participants from both the polluted and control areas. According to the urinary tests, subjects were divided into 4 groups as follows: a group with neither proteinuria nor glucosuria (no finding group), a group with glucosuria without proteinuria (glucosuria group), a group with proteinuria without glucosuria (proteinuria group), and a group with both glucosuria and proteinuria (glucoproteinuria group), defined with cut-off values of protein ≥ 10 mg/dl and glucose ≥ +/-. Prevalence of each urinary finding in the Cd-polluted and control areas are shown in Table 1 that includes the smoking rate at the examination and history of hypertension. At this time, information regarding health status and lifestyle were obtained from the questionnaire of the health survey in 1979–1984 [9]. Urinary protein was quantified using the Kingsbury-Clark method, and qualitative tests using strips were performed to detect urinary glucose levels.

**Table 1:** Characteristic differences among four groups according to urinary findings in controls and exposed subjects

	**Pro(-) and Glu(-)**		**Pro(-) and Glu(+)**		**Pro(+) and Glu(-)**		**Pro(+) and Glu(+)**	
	**N**	**Rate, %**	**N**	**Rate, %**	**N**	**Rate, %**	**N**	**Rate, %**
*Men*								
Controls	n = 748	(80.8%)	n = 143	(15.4%)	n = 27	(2.9%)	n = 8	(0.9%)
Smokers	437	58.4	94	65.7	18	66.7	5	62.5
HT history	175	23.4	38	26.6	10	26.6	6	75.0
Exposed subjects	n = 2556	(76.8%)	n = 498	(14.8%)	n = 150	(4.5%)	n = 159	(4.7%)
Smokers	1592	62.3	311	62.4	85	56.7	89	60.0
HT history	551	21.6	101	20.3	45	30.0	39	24.5
*Women*								
Controls	n = 1039	(88.7%)	n = 83	(7.1%)	n = 44	(3.8%)	n = 6	(0.5%)
Smokers	26	2.5	1	1.2	1	2.3	0	0.0
HT history	254	24.4	24	28.9	17	38.6	1	16.6
Exposed subjects	n = 3184	(79.9%)	n = 398	(10.0%)	n = 136	(3.4%)	n = 267	(6.7%)
Smokers	257	82.4	23	7.4	7	2.2	25	8.0
HT history	650	76.7	98	11.6	41	4.8	58	6.8

Pro(+): urinary protein ≥ 10 mg/dL, Pro(-): urinary protein <10 mg/dL, Glu(+): urinary glucose ≥ +/-, Glu(-): urinary glucose negative.

n: number of all subjects in each group, N: number of subjects, (%): rate per total.

In the second step of the screening survey, only subjects positive for urinary protein and/or glucose in the Cd-polluted Jinzu River basin were examined for their urinary levels of Cd, ß2-micloglobuline (MG), lysozyme (LTZ) and total amino acids (amino) in the same urine specimen to confirm that these subjects with urinary findings have increased Cd and renal tubular dysfunction indicated by these markers (Table S1 in an Additional file shows details [see Additional file 1]). These results suggest that urinary Cd levels in the exposed area were higher than those in controls; geometrical mean (geometrical standard) = 4.0 (1.9) μg/L for men and 4.4 (2.2) for women [9], measured by ammonium pyrrolidine dithiocarbamate (APDC) – methyl isobutyl ketone (MIBK) extraction atomic absorption spectrometry in Toyama Institute of Health [10]. The subjects in the glucoproteinuria group were suspected to have renal tubular dysfunction because of high positive rates of urinary low molecular protein, such as ß2-MG and/or LTZ measured by immunoelectrophoresis and the lysoplate method, respectively.

### A follow-up survey

Permission was obtained from a regional Legal Affairs Bureau in June 2005 to use family registry records for scientific purposes. We collected family registry records of all subjects from each city office and determined their survival status (alive or dead) as of November 30, 2005 and the date of death if applicable. The address at the time of the health survey was considered as the family registry address. Cause of death was determined from vital statistics data by linking it to health survey data with survival status based on birthday, death day, gender, and address after obtaining permission from the Ministry of Health, Labour and Welfare to use vital statistics for other research purposes on August 12, 2009.

The Ethics Committee of Kanazawa Medical University in Uchinada, Ishikawa 920–0293, Japan, where the analysis of the data was conducted, approved the present study.

According the follow-up survey, following results were obtained; a total of 166 subjects (1.7%; 133 exposed and 33 controls) were lost to follow-up during the observation period because their status could not be determined. In all areas, 5351 deaths occurred. The cause of death was determined for 5276 cases (98.5%) by successful data linkage and classified according to the 9th Revised International Classification of Diseases (ICD 9) from 1979 to 1994 and the ICD 10 from 1995 to 2005 (Table S2 in an Additional file shows more details [see Additional file 1]).

### Mortality analysis

To determine the survival period of each subject, the health survey date was used as the starting point and the life status survey date, November 30, 2005, was considered the end of follow-up for living subjects. A total of 232 subjects, including 157 cases with unknown life status and 75 cases with unknown causes of death were excluded from the analysis. Mortalities of all subjects in the polluted area and controls were calculated by the person-years method for all 26 years from baseline in 1979 to November 30, 2005.

For standardized mortality analysis for all causes, standardized mortality ratio (SMR) was calculated after adjusting age using age distribution and death rates per decade of the general Japanese population during the observation period as a standard population. Subsequently, analysis of the risk of mortality after adjustment for age, smoking status at present (yes: 1, no: 0), and history of hypertension (yes: 1, no: 0) was conducted using Cox’s proportional hazard model in each gender to compare 5 groups of 4 exposed groups with different urinary findings and 1 control group with urinary protein positive and/or glucose positive findings, with the control no finding group. At this time, controls in the glucosuria group, proteinuria group, glucoproteinuria group were set into one group (with findings) because of a small number of subjects for mortality analysis. All analyses were performed using SPSS 16.0 software (SPSS Inc., Chicago, IL, USA). P values of <0.05 were considered statistically significant.

## Results

Table 2 shows the SMRs and hazard ratios (mortality risk ratios) in the groups of both genders with different urinary findings in the control and Cd-polluted areas. There is a generally higher mortality in the Cd polluted area compared with the control area. In men, the SMRs of the control group with urinary findings and the exposed glucoproteinuria group in the Cd-exposed area were significantly higher than that in the general Japanese population, whereas the SMRs of the no finding groups were significantly lower in both the exposed and control areas. The mortality risk ratios for all causes in the proteinuria and glucoproteinuria groups of exposed men were 1.27 and 1.57, respectively, and significantly increased compared with the no finding group in the control area. Similarly, the mortality risk ratio of the control group with urinary findings was also significantly increased compared with the no finding group in the controls.

**Table 2:** The standardized mortality ratios and hazard ratios in different groups in controls and exposed subjects

**Area**	**Urinary findings**	**Groups**	**N**	**PY**	**PY/person**	**D**	**SMR**	**(95% CI)**	**Hazard ratio**	**(95% CI)**
*Men*										
Control	Pro(-) and Glu(-)	No finding	748	12514	16.7	441	0.88	(0.80, 0.96)	1.00	
	Pro(+) and/or Glu(+)	With findings	178	2608	14.7	118	1.24	(1.01, 1.46)	1.39	(1.13, 1.70)**
Exposed	Pro(-) and Glu(-)	No finding	2556	44636	17.5	1595	0.90	(0.85, 0.94)	1.02	(0.92, 1.14)
	Pro(-) and Glu(+)	Glucoseuria	498	8444	17.0	331	0.96	(0.86, 1.07)	1.10	(0.95, 1.27)
	Pro(+) and Glu(-)	Proteinuria	150	1905	12.7	123	1.19	(0.98, 1.40)	1.27	(1.03, 1.57)*
	Pro(+) and Glu(+)	Glucoproteinuria	159	1579	9.9	147	1.40	(1.17, 1.63)	1.57	(1.29, 1.90)***
*Women*										
Control	Pro(-) and Glu(-)	No finding	1039	19085	18.4	455	0.89	(0.81, 0.97)	1.00	
	Pro(+) and/or Glu(+)	with findings	133	1884	14.2	77	1.16	(0.90, 1.41)	1.26	(0.99, 1.61)
Exposed	Pro(-) and Glu(-)	No finding	3184	64520	20.3	1405	0.84	(0.90, 0.88)	0.93	(0.83, 1.03)
	Pro(-) and Glu(+)	Glucoseuria	398	6568	16.5	278	1.27	(1.12, 1.41)	1.40	(1.20, 1.63)***
	Pro(+) and Glu(-)	Proteinuria	136	1947	14.3	106	1.44	(1.16, 1.71)	1.77	(1.42, 2.20)***
	Pro(+) and Glu(+)	Glucoproteinuria	267	2406	9.0	257	2.02	(1.77, 2.26)	2.40	(2.16, 2.80)***

Pro(+): urinary protein ≥ 10 mg/dL, Pro(-): urinary protein <10 mg/dL, Glu(+): urinary glucose ≥ +/-, Glu(-): urinary glucose negative.

*N* number of subjects, *PY* person year, *D* number of death, *SMR* standardized mortality ratio.

*CI* confidence interval, hazard ratio adjusted with age, smoking status, and history of hypertension.

*P < 0.05, **P < 0.01, ***P < 0.001 compared with Pro(-) and Glu(-) in controls.

In women, the SMRs were significantly higher in the exposed glucosuria, proteinuria, and glucoproteinuria groups compared with the general Japanese population, but not in the control group with urinary findings (Table 2). The mortality risk ratios for all causes in these three exposed groups, glucosuria, proteinuria, and glucoproteinuria groups, were 1.40, 1.77, and 2.40, respectively, and were significantly increased compared with the control no finding group, whereas increased mortality risk ratio was not significant in the control group with urinary findings (Table 2).

The number of deaths and mortality risk ratios for specific causes of deaths in the four exposed groups and one control group with urinary findings in men compared with the control no finding group are shown in Tables 3 and 4. In men, no significant increased mortality risk ratio for malignant neoplasms were found in any exposed and control groups. The mortality risk ratios for endocrine, nutritional and metabolic diseases in the exposed glucosuria group and the glucoproteinuria group in the exposed areas and control group with urinary findings were 3.42, 5.80, and 6.80, respectively, and were significantly increased compared with the control no finding group. At this time, all deaths from endocrine, nutritional, and metabolic diseases in these groups except the control no finding group were from diabetes (Table 3).

**Table 3:** Hazard ratios for cancer and diabetes in the groups with different urinary findings in men

**Cause of deaths**	**Control areas**					**Cd polluted areas**											
	**Pro(-) and Glu(-)**		**Pro(+) and/or Glu(+)**			**Pro(-) and Glu(-)**			**Pro(-) and Glu(+)**			**Pro(+) and Glu(-)**			**Pro(+) and Glu(+)**		
	**(N=748)**		**(N=178)**			**(N=2556)**			**(N=331)**			**(N=123)**			**(N=147)**		
	**D**	**HR**	**D**	**HR**	**(95% CI)**	**D**	**HR**	**(95% CI)**	**D**	**HR**	**(95% CI)**	**D**	**HR**	**(95% CI)**	**D**	**HR**	**(95% CI)**
Malignant neoplasms	142	1.00	32	1.16	(0.79, 1.71)	522	1.05	(0.87, 1.27)	82	0.87	(0.66, 1.14)	26	0.97	(0.62, 1.51)	23	1.04	(0.66, 1.63)
Esophagus	2	1.00	2	4.58	(0.64, 32.6)	20	2.63	(0.61, 11.3)	6	3.59	(0.70, 18.5)	1	2.94	(0.26, 32.8)	0	-	-
Stomach	48	1.00	9	1.02	(0.50, 2.09)	142	0.88	(0.63, 1.25)	26	0.87	(0.53, 1.42)	6	0.66	(0.26, 1.68)	6	0.80	(0.34, 1.90)
Colon, rectum	14	1.00	2	0.68	(0.15, 2.98)	53	1.01	(0.55, 1.82)	7	0.73	(0.29, 1.80)	2	0.85	(0.19, 3.78)	2	0.99	(0.22, 4.41)
Liver	15	1.00	1	0.35	(0.05, 2.64)	42	0.82	(0.44, 1.51)	8	0.84	(0.35, 2.01)	5	2.27	(0.81, 6.36)	3	1.59	(0.45, 5.61)
Gall bladder	6	1.00	1	0.93	(0.11, 7.70)	21	1.02	(0.41, 2.55)	4	1.01	(0.29, 3.60)	0	-	-	0	-	-
Pancreas	7	1.00	2	1.76	(0.35, 8.71)	33	1.45	(0.60, 3.48)	6	1.40	(0.45, 4.35)	0	-	-	2	2.16	(0.42, 11.0)
Lung	30	1.00	7	1.13	(0.50, 2.57)	121	1.08	(0.72, 1.62)	11	0.52	(0.26, 1.03)	5	0.84	(0.32, 2.18)	6	1.19	(0.49, 2.89)
Prostate	3	1.00	2	3.81	(0.63, 22.9)	13	1.16	(0.33, 4.09)	5	2.32	(0.55, 9.74)	3	1.43	(0.15, 14.2)	0	-	-
Bladder	3	1.00	0	-	-	11	1.03	(0.28, 3.72)	1	0.48	(0.05, 4.58)	1	1.87	(0.19, 18.3)	0	-	-
Kidney and urinal tract	1	1.00	1	5.00	(0.31, 80.4)	5	1.23	(0.14, 11.1)	0	-	-	2	11.0	(0.96, 125)	1	4.39	(0.25, 76.9)
Lymph, blood forming organs	6	1.00	0	-	-	24	1.13	(0.46, 2.78)	4	0.97	(0.27, 3.45)	1	1.05	(0.13, 8.82)	2	2.27	(0.44, 11.6)
Endocrine, nutritional and metabolic diseases	4	1.00	5	6.81	(1.82, 25.5)**	15	0.89	(0.29, 2.71)	11	3.42	(1.08, 10.8)*	0	-	-	4	5.80	(1.39, 24.2)*
Diabetes	1	1.00	5	27.8	(3.23, 238)**	11	2.56	(0.33, 20.0)	11	13.4	(1.72, 105)*	0	-	-	4	23.3	(2.53, 214)**

Pro(+): urinary protein ≥ 10 mg/dL, Pro(-): urinary protein <10 mg/dL, Glu(+): urinary glucose ≥ +/-, Glu(-): urinary glucose negative.

*N* number of subjects, *D* number of deaths, *HR* Hazard ratio, *CI* confident interval.

*P < 0.05, **P < 0.01 compared with Pro(-) and Glu(-) in controls.

**Table 4:** Hazard ratios for the other diseases in the groups with different urinary findings in men

**Cause of deaths**	**Control areas**					**Cd polluted areas**											
	**Pro(-) and Glu(-)**		**Pro(+) or/and Glu(+)**			**Pro(-) and Glu(-)**			**Pro(-) and Glu(+)**			**Pro(+) and Glu(-)**			**Pro(+) and Glu(+)**		
	**(N=748)**		**(N=178)**			**(N=2556)**			**(N=331)**			**(N=123)**			**(N=147)**		
	**D**	**HR**	**D**	**HR**	**(95% CI)**	**D**	**HR**	**(95% CI)**	**D**	**HR**	**(95% CI)**	**D**	**HR**	**(95% CI)**	**D**	**HR**	**(95% CI)**
Cardiovascular diseases	61	1.00	20	1.61	(0.96, 2.69)	255	1.20	(0.91, 1.60)	51	1.18	(0.81, 1.73)	23	1.55	(0.95, 2.52)	31	1.81	(1.16, 2.85)*
Ischemic Heart diseases	20	1.00	7	1.77	(0.75, 4.19)	112	1.65	(1.02, 2.66)*	16	1.12	(0.57, 2.19)	5	1.02	(0.38, 2.07)	14	2.76	(1.35, 5.67)**
Heart failure	28	1.00	11	1.84	(0.89, 3.81)	98	1.00	(0.65, 1.54)	28	1.46	(0.85, 2.49)	14	2.00	(1.04, 3.86)*	16	1.67	(0.87, 3.20)
Cerebrovascular diseases	68	1.00	16	1.23	(0.71, 2.13)	207	0.90	(0.68, 1.19)	53	1.14	(0.79, 1.66)	21	1.19	(0.71, 1.99)	16	0.94	(0.54, 1.65)
Cerebral hemorrage	20	1.00	4	1.05	(0.36, 3.11)	33	0.48	(0.27, 0.87)*	11	0.91	(0.43, 1.92)	8	2.31	(0.99, 5.38)	0	-	-
Cerebral infarction	41	1.00	11	1.47	(0.75, 2.87)	130	0.94	(0.65, 1.34)	36	1.28	(0.81, 2.03)	6	0.49	(0.19, 1.24)	12	1.22	(0.63, 2.37)
Subarachnoid hemorrhage	4	1.00	0	-	-	11	0.68	(0.22, 2.17)	2	0.32	(0.04, 2.88)	2	2.74	(0.49, 15.3)	0	-	-
Diseases of the respiratory systems	77	1.00	22	1.53	(0.94, 2.48)	301	1.11	(0.86, 1.43)	63	1.22	(0.87, 1.71)	27	1.44	(0.92, 2.27)	30	1.64	(1.06, 2.54)*
Diseases of the digestive systems	14	1.00	3	1.17	(0.33, 4.12)	55	1.19	(0.65, 2.19)	17	1.84	(0.88, 3.83)	3	1.13	(0.32, 4.00)	7	2.38	(0.93, 6.13)
Kidney and urinal tract diseases	5	1.00	3	3.15	(0.75, 13.2)	28	1.51	(0.58, 3.94)	5	1.47	(0.43, 5.10)	6	5.60	(1.68, 18.7)**	11	9.51	(3.14, 28.8)***
Renal diseases	4	1.00	3	3.91	(0.87, 17.5)	23	1.59	(0.54, 4.62)	5	1.85	(0.50, 6.90)	6	7.01	(1.94, 25.3)**	10	12.4	(3.77, 41.1)***
External causes of mortality	39	1.00	9	1.22	(0.59, 2.52)	85	0.58	(0.39, 0.85)**	12	0.58	(0.32, 1.06)	3	0.46	(0.14, 1.48)	13	2.32	(1.23, 4.38)*
Accident	29	1.00	5	0.91	(0.35, 2.35)	68	0.60	(0.39, 0.95)*	12	0.61	(0.31, 1.20)	3	0.57	(0.17, 1.88)	13	2.69	(1.36, 5.34)**
Accident except Cd toxicity	29	1.00	5	0.91	(0.35, 2.36)	68	0.60	(0.39, 0.95)*	12	0.61	(0.31, 1.21)	3	0.59	(0.18, 1.94)	8	1.76	(0.78, 3.94)
Toxic effects of cadmium	0	1.00	0	-	-	0	-	-	0	-	-	0	-	-	5	-	-

Pro(+): urinary protein ≥ 10 mg/dL, Pro(-): urinary protein <10 mg/dL, Glu(+): urinary glucose ≥ +/-, Glu(-): urinary glucose negative.

*N* number of subjects, *D* number of deaths, *HR* Hazard ratio, *CI* confident interval, Cd toxicity = Toxic effect of cadmium.

*P < 0.05, **P < 0.01, ***P < 0.001 compared with Pro(-) and Glu(-) in controls.

Regarding cardiovascular diseases in men, the mortality risk ratio for ischemic heart disease was significantly increased in the glucosuria and glucoproteinuria groups, and the risk for heart failure was higher in the exposed proteinuria group (Table 4). Diseases of the respiratory systems showed increased mortality risk only in the male exposed glucoproteinuria group. Particularly, the mortality risk ratio for renal diseases, a main disease at the subcategory of kidney and urinary tract diseases, of the exposed glucoproteinuria group was 12.4 in men and a very high value. Similarly, renal diseases also showed a high mortality risk ratio in the male exposed proteinuria group. In the male exposed glucoproteinuria group, the mortality risk ratio for external causes, particularly by accident, was significantly increased compared with the male control no finding group because the five deaths from toxic effects of Cd included deaths by accident (Table 4). In contrast, the mortality risk ratio for external causes in the male exposed no finding group was significantly lower compared with the control no finding group.

Tables 5 and 6 also show hazard ratios for specific causes of four exposed groups and one control group with urinary findings compared with the control no finding group in women. The mortality risk ratios for all malignant neoplasms in the female exposed proteinuria and glucoproteinuria groups were 1.97 and 1.64, respectively, and significantly increased compared with the control no finding group (Table 5). Colorectal cancer, uterine cancer, and kidney and urinary tract cancer in the exposed glucoproteinuria group, and breast cancer, uterine cancer, and kidney and urinary tract cancer in the exposed proteinuria group showed a significant increase of mortality risk ratios in women. Increased mortality risk ratios for gall bladder cancer in the exposed glucosuria group and for colorectal cancer in the exposed no finding group were found in women (Table 5). Mortality risk ratios for diabetes were significantly increased in the exposed glucosuria and glucoproteinuria groups in women (Table 5).

**Table 5:** Hazard ratios for cancer and diabetes in the groups with different urinary findings in women

**Cause of deaths**	**Control areas**					**Polluted areas**											
	**Pro(-) and Glu(-)**		**Pro(+) or/and Glu(+)**			**Pro(-) and Glu(-)**			**Pro(-) and Glu(+)**			**Pro(+) and Glu(-)**			**Pro(+) and Glu(+)**		
	**(N=1039)**		**(N=133)**			**(N=3184)**			**(N=398)**			**(N=136)**			**(N=267)**		
	**D**	**HR**	**D**	**HR**	**(95% CI)**	**D**	**HR**	**(95% CI)**	**D**	**HR**	**(95% CI)**	**D**	**HR**	**(95% CI)**	**D**	**HR**	**(95% CI)**
Malignant neoplasm	97	1.00	17	1.57	(0.94, 2.64)	339	1.03	(0.82, 1.31)	51	1.34	(0.95, 1.90)	21	1.97	(1.21, 3.19)**	26	1.64	(1.05, 2.56)*
Esophagus	0	1.00	0	-	-	2	-	-	2	-	-	0	-	-	0	-	-
Stomach	26	1.00	7	2.29	(0.99, 5.34)	77	0.99	(0.62, 1.58)	11	1.12	(0.55, 2.30)	5	1.42	(0.49, 4.09)	5	0.94	(0.35, 2.51)
Colon, rectum	7	1.00	1	1.14	(0.14, 9.33)	55	2.42	(1.10, 5.34)*	5	1.77	(0.56, 5.59)	0	-	-	6	4.27	(1.39, 13.1)*
Liver	5	1.00	0	-	-	4	.54	(0.52, 4.54)	2	0.70	(0.08, 6.27)	1	2.59	(0.29, 23.3)	0	-	-
Gall bladder	7	1.00	1	1.28	(0.16, 10.4)	36	1.44	(0.63, 3.28)	8	3.21	(1.16, 8.90)*	1	1.40	(0.17, 11.4)	1	1.09	(0.13, 9.09)
Pancreas	12	1.00	2	1.60	(0.35, 7.24)	25	0.60	(0.29, 1.24)	4	0.89	(0.28, 2.82)	3	2.39	(0.66, 8.62)	2	1.03	(0.22, 4.85)
Lung	17	1.00	2	1.03	(0.24, 4.50)	35	0.50	(0.28, 0.92)*	4	0.56	(0.19, 1.67)	2	1.01	(0.23, 4.38)	3	1.04	(0.29, 3.67)
Breast	4	1.00	0	-	-	11	0.76	(0.24, 2.44)	1	0.80	(0.09, 7.19)	2	5.73	(1.04, 31.4)*	0		
Uterus	5	1.00	1	4.56	(0.41, 50.6)	10	1.15	(0.24, 5.57)	0			2	10.5	(1.46, 75.1)*	3	10.8	(1.62, 71.7)*
Ovary	5	1.00	0	-	-	7	1.17	(0.24, 5.67)	2	2.73	(0.38, 19.6)	0	-	-	0		
Bladder	1	1.00	1	6.72	(0.40, 112)	4	1.21	(0.13, 11.2)	0	-	-	0	-	-	0	-	-
Kidney and urinal tract	1	1.00	0	-	-	3	0.82	(0.08, 8.06)	1	2.61	(0.16, 42.1)	2	19.4	(1.73, 217)*	2	15.5	(1.25, 192)*
Lymph, blood forming organs	4	1.00	1	2.46	(0.27, 22.0)	20	1.38	(0.46, 4.08)	3	2.10	(0.47, 9.46)	2	5.11	(0.93, 28.1)	2	4.56	(0.79, 26.1)
Endocrine, nutritional and metabolic diseases	3	1.00	1	2.61	(0.27, 25.2)	14	1.37	(0.39, 4.84)	11	8.58	(2.38, 30.9)***	1	2.70	(0.28, 26.1)	4	6.67	(1.44, 31.0)*
Diabetes	2	1.00	1	4.11	(0.37, 45.6)	6	0.81	(0.16, 4.09)	11	12.7	(2.80, 57.8)***	1	4.21	(0.38, 46.8)	4	10.0	(1.73, 57.8)**

Pro(+): urinary protein ≥ 10 mg/dL, Pro(-): urinary protein <10 mg/dL, Glu(+): urinary glucose ≥ +/-, Glu(-): urinary glucose negative.

*N* number of subjects, *D* number of deaths, *HR* Hazard ratio, *CI* confident interval.

*P < 0.05, **P < 0.01, ***P < 0.001 compared with Pro(-) and Glu(-) in controls.

**Table 6:** Hazard ratios for the other diseases in the groups with different urinary findings in women

**Cause of deaths**	**Control areas**					**Cd polluted areas**											
	**Pro(-) and Glu(-)**		**Pro(+) or/and Glu(+)**			**Pro(-) and Glu(-)**			**Pro(-) and Glu(+)**			**Pro(+) and Glu(-)**			**Pro(+) and Glu(+)**		
	**(N=1039)**		**(N=133)**			**(N=3184)**			**(N=398)**			**(N=136)**			**(N=267)**		
	**D**	**HR**	**D**	**HR**	**(95% CI)**	**D**	**HR**	**(95% CI)**	**D**	**HR**	**(95% CI)**	**D**	**HR**	**(95% CI)**	**D**	**HR**	**(95% CI)**
Cardiovascular diseases	90	1.00	13	0.98	(0.55, 1.77)	271	0.95	(0.74, 1.22)	54	1.36	(0.96, 1.92)	20	1.48	(0.89, 2.46)	52	2.02	(1.40, 2.91)***
Ischemic Heart diseases	23	1.00	4	1.49	(0.51, 4.37)	127	1.64	(1.01, 2.66)*	21	2.32	(1.25, 4.33)**	5	1.53	(0.52, 4.49)	9	3.79	(1.95, 7.39)***
Heart failure	47	1.00	6	0.75	(0.32, 1.77)	111	0.77	(0.54, 1.10)	27	1.23	(0.76, 1.99)	10	1.30	(0.63, 2.67)	27	1.71	(1.03, 2.83)*
Cerebrovascular diseases	89	1.00	20	1.50	(0.92, 2.44)	236	0.88	(0.68, 1.13)	43	1.09	(0.75, 1.58)	21	1.70	(1.04, 2.77)*	29	1.21	(0.78, 1.87)
Cerebral hemorrage	19	1.00	4	1.64	(0.56, 4.86)	45	0.70	(0.41, 1.22)	3	0.38	(0.11, 1.30)	4	1.70	(0.58, 5.02)	4	0.77	(0.22, 2.68)
Cerebral infarction	54	1.00	14	1.75	(0.97, 3.17)	142	0.92	(0.66, 1.27)	28	1.16	(0.73, 1.86)	9	1.31	(0.65, 2.67)	14	1.09	(0.60, 1.99)
Subarachnoid hemorrhage	7	1.00	1	1.16	(0.14, 9.43)	20	0.95	(0.40, 2.27)	3	1.15	(0.30, 4.47)	3	3.94	(1.01, 15.4)*	1	0.75	(0.09, 6.33)
Diseases of the respiratory systems	64	1.00	4	0.43	(0.16, 1.19)	205	1.01	(0.75, 1.36)	35	1.23	(0.80, 1.88)	11	1.22	(0.62, 2.38)	27	1.75	(1.10, 2.78)*
Diseases of the digestive systems	16	1.00	2	0.93	(0.21, 4.10)	53	1.02	(0.57, 1.84)	18	2.64	(1.32, 5.29)**	4	2.12	(0.70, 6.39)	11	2.96	(1.33, 6.60)**
Kidney and urinal tract diseases	8	1.00	5	4.06	(1.22, 13.5)*	38	1.35	(0.63, 2.91)	20	5.86	(2.57, 13.3)***	8	7.10	(2.57, 19.6)***	30	20.1	(8.99, 44.8)***
Renal diseases	7	1.00	5	4.78	(1.39, 16.4)*	32	1.33	(0.59, 3.03)	17	5.73	(2.37, 13.9)***	8	8.31	(2.91, 23.7)***	29	23.2	(9.92, 54.3)***
External causes of mortality	35	1.00	2	0.47	(0.11, 1.95)	75	0.60	(0.40, 0.91)*	10	0.91	(0.48, 1.73)	5		(0.62, 3.53)	49	7.01	(4.41, 11.1)***
Accident	21	1.00	1	0.40	(0.05, 2.97)	56	0.75	(0.45, 1.25)	10	1.17	(0.55, 2.49)	5	2.07	(0.78, 5.51)	49	12.3	(7.16, 21.2)***
Accident except Cd toxicity	21	1.00	1	0.44	(0.06, 3.28)	55	0.70	(0.42, 1.17)	5	0.60	(0.23, 1.59)	5	2.16	(0.81, 17.3)	4	1.28	(0.43, 3.84)
Toxic effects of cadmium	0	1.00	0	-	-	1	-	-	5	-	-	0	-	-	45	-	-

Pro(+): urinary protein ≥ 10 mg/dL, Pro(-): urinary protein <10 mg/dL, Glu(+): urinary glucose ≥ +/-, Glu(-): urinary glucose negative.

*N* number of subjects, *D* number of deaths, *HR* Hazard ratio, *CI* confident interval, Cd toxicity = Toxic effect of cadmium.

*P < 0.05, **P < 0.01, ***P < 0.001 compared with Pro(-) and Glu(-) in controls.

In women, the exposed glucoproteinuria group showed significantly increased mortality risk ratios for cardiovascular diseases, especially ischemic heart disease, diseases of the respiratory system, diseases of the digestive system, renal diseases, and external causes of deaths including accidents (Table 6). Mortality risk ratios for cerebrovascular diseases and renal diseases of the exposed proteinuria group and for ischemic heart diseases, digestive diseases and renal diseases of the exposed glucosuria group were significantly increased in women (Table 6). The mortality risk ratio for ischemic heart diseases was also significantly increased in the exposed no finding group in women. In addition, the mortality risk ratio for renal diseases was significantly increased in the control group with urinary findings as well as the exposed groups with urinary findings in women (Table 6).

## Discussion

### Causes of deaths and renal tubular dysfunction

In the present study, mortality risks of the exposed glucoproteinuria groups suspected of renal tubular dysfunction due to increased urinary β2-MG and/or LTZ were significantly increased in both genders. In the dead subjects of glucoproteinuria group, 5 men (3.4%) and 45 women (17.5%) died from toxic effects of cadmium (Cd toxicosis), which were classified into accidents. Causes of deaths contributed to their increased mortality other than Cd toxicosis were respiratory diseases, renal diseases, cardiovascular diseases, and diabetes in men, and all diseases except cerebrovascular diseases in women. In the Cd-exposed residents in the Jinzu River basin, the prevalence of subjects with glucoproteinuria was 4.7% in men and 6.7% in women and much higher than that in controls (0.9% in men, and 0.5% in women), suggesting that environmental Cd exposure increased the number of renal tubular dysfunction cases indicated by glucoproteinuria and characterized by an unfavorable long-term life prognosis. Particularly in women, health effects caused by Cd exposure cover almost all diseases to shorten the life span.

Although the prevalence of the exposed glucosuria and proteinuria groups were similar to those of the glucosuria and proteinuria groups in controls in both genders, mortality risks of these exposed groups, particularly the proteinuria group, were higher than that in the control group with urinary findings in women. Regarding the causes of deaths, mortality risks for malignant neoplasms and cerebrovascular diseases of the exposed proteinuria group were increased, whereas the increased risks of the control group with urinary findings were not significant in women. These results suggest that the effect of Cd exposure on mortality was exhibited in women with only proteinuria who might include renal tubular dysfunction cases because 40% of them showed low molecular proteinuria at the second screening tests at baseline.

### Toxic effects of Cd (Cd toxicosis and itai-itai diseases) and renal diseases

The mortality risk ratios for accidents including the toxic effects of Cd were high, but they were not high for accidents without Cd toxic effects in the glucoproteinuria groups in both genders, suggesting that Cd toxicosis and itai-itai disease significantly increased the mortality of residents with renal tubular dysfunction. Itai-itai disease involves suffering from bone pain and renal tubular dysfunctions, and renal damage is pathologically characterized by renal atrophy due to an advanced decrease in renal tubuli [7], which leads to renal failure after long-term observation [11]. Therefore, mortality risk for renal diseases in the glucoproteinuria groups might be underestimated because they were diagnosed as itai-itai disease, not renal diseases even if they had renal failure. Consistently, in the first 10 years observation, the numerous deaths due to itai-itai diseases were observed in all polluted subjects without increased mortality from renal diseases (Table S1 in an Additional file shows this in more detail [see Additional file 2]).

Since Lauwers and De Wals in Belgium suggested that increased mortality rates from renal disease were caused by environmental Cd exposure, renal diseases have been an important cause of death among inhabitants living in Cd-polluted areas [12]. In the Cd-polluted Kakehashi River basin in Japan, we have reported higher mortality risks for renal diseases among subjects with β_2_-MG ≥1000 μg/g Cr in both sexes [6,13] and urinary Cd ≥10 μg/g Cr in women [14,15]. In the present study, significantly increased mortality risks for kidney and urinary tract diseases, particularly renal diseases including renal failure, were observed in the exposed two groups with proteinuria group in men, and in the three exposed groups with glucosuria and/or proteinuria in women. Similarly, renal disease risk of the control group with urinary findings was significantly increased in women, suggesting that women with glucosuria and/or proteinuria induced by any causes are at risk for fatal renal diseases. However, the mortality risk ratio for renal diseases in the exposed glucoproteinuria group was 3-fold higher in men and 5-fold higher in women, suggesting that renal dysfunction induced by Cd may enhance deterioration of renal function in these groups.

### Cancer mortality

Mortality because of cancer is a focal research concern because Cd and its compounds were classified as a human carcinogen by the International Agency for Research on Cancer [1,2]. Several follow-up surveys in factory workers have been conducted [5,16-18], and results suggest that mortality increases because of renal diseases and malignant neoplasms, such as lung cancer and prostate cancer. In a study of inhabitants exposed to environmental Cd in Belgium, an increased risk of lung cancer was reported in those living in high exposure areas compared with less polluted areas [4]. However, in the present study, the risk of mortality for lung cancer in the exposed subjects was not higher in men and was even significantly lower in women. The Cd exposure route of the factory workers and inhabitants of the Belgian polluted area was different from our subjects in Japan. Aspiration of dust in workers and Belgian inhabitants and oral intake in Japanese inhabitants may be the cause of inconsistencies in the results for lung cancer risk. An increased mortality risk for prostatic cancer was also not found in the present study.

In our study, increased colorectal cancer mortality risk was observed in women with glucoproteinuria in exposed areas. This is a new finding that has never been reported in any previous epidemiologic studies for factory workers and residents in Cd-polluted areas. However, mortality risk for colorectal cancer also increased in women without urinary findings in the polluted area, indicating an association of colorectal cancer mortality with Cd exposure, not renal tubular dysfunction. In fact, mortality risk for colorectal cancer significantly increased in all female exposed subjects compared with all controls in the present study, and we have reported this evidence in our previous study report (Table S2 in an Additional file shows this in more detail [see Additional file 2]). Recently, an inverse association between 25-hydroxy (OH)-vitamin D (Vit. D) and colorectal cancer was identified in a large-scale epidemiologic study of a western European population [19]. Feskanich et al. also reported that women in the highest quintile of 1,25-(OH)_2_-Vit. D had a higher risk for colorectal cancer [20]. In the Jinzu River basin, Aoshima et al. investigated serum 25-OH-Vit. D and 1,25-(OH)_2_-Vit. D levels in inhabitants with relatively maintained glomerular function and found that only 1,25-(OH)_2_-Vit. D levels were higher than the upper range of the reference value because of compensatory secretion of parathyroid hormone, particularly in women [21]. These effects of Cd exposure on 1,25-(OH)_2_-Vit. D may increase the risk of colorectal cancer in women living in the Jinzu River basin. However, a further study is necessary to clarify the mechanisms of association between colorectal cancer and Cd exposure.

In the exposed proteinuria group and glucoproteinuria group in women, mortality risks for kidney and urinary tract cancer were significantly increased in women. Mortality risk ratios for this cancer were also high in men (11.0, proteinuria group; 4.39, glucoproteinuria group), although their increases were not statistically significant. Ilyasova and Schwartz performed a systemic review of the literature on cadmium and renal cancer and reported that exposure to cadmium appears to be associated with renal cancer [22]. These results suggest that renal damage induced by Cd may be a risk for kidney and urinary tract cancer.

Mortality risks for uterine cancer in the exposed proteinuria group and glucoproteinuria group and for breast cancer in the exposed proteinuria group were higher in women, albeit the numbers of deaths were small. Regarding uterine cancer, Akesson et al. reported that dietary Cd intake increased postmenopausal endometrial cancer incidence in a Swedish cohort [23], suggesting estrogen-mimicking effects of Cd exposure in women. For breast cancer, Nishijo et al. reported an increased mortality risk of breast cancer in women with urinary β2-MG ≥1000 μg/g Cr during the first 5 and 10 years of observation of Cd-exposed inhabitants in the Kakehashi River basin, Japan [6]. In the U.S. population, McElroy et al. reported increased breast cancer risk with increasing urinary Cd levels based on a case–control study [24]. Julin et al. performed a cohort study of the Swedish population and found associations between dietary Cd and breast cancer incidence [25]. Collectively, these study results suggest that Cd exposure increases the risks of hormone-related cancers, particularly in women.

### Circulatory diseases, respiratory diseases and digestive diseases

In the present study, significant increased mortality risk for ischemic cardiovascular diseases was observed in the exposed glucoproteinuria group and no finding group in men and in the three exposed groups other than the proteinuria group in women. Previously, we have reported that the risk of mortality for ischemic heart diseases was significantly higher in all women living in the polluted areas than that in controls (Table S2 in an Additional file shows this in more detail [see Additional file 2]), and in the present study, we found that mortality risk ratios for this disease were higher particularly in the exposed glucoproteinuria group in both sexes (2.76, men; 3.79, women). In addition, we focused on the subjects who didn’t smoke and had no diabetes, and analyzed their mortality from ischemic heart diseases (Table S3 and S4 in an Additional file show more details [see Additional file 1]), because statistical adjustment of effects of smoking status on mortality might be not sufficient and diabetes might influence mortality rather than direct Cd exposure. However, even after excluding the diabetes and smokers, significant increased mortality risk ratios from ischemic heart diseases were exhibited in both genders. In the U.S. population, Menke et al. found that urinary Cd was associated with an increased risk of cardiovascular mortality, particularly coronary vascular diseases, in men who participated in the 1988–1991 United States National Health and Nutrition Examination Survey (NHANES) [26], and Tellez-Plaza et al. also reported an association of Cd exposure and cardiovascular mortality in both men and women who were participants in the 1999–2004 NHANES survey [27]. These results are concordant with our results, albeit their exposure level was at background. In Shipham, a Cd-polluted area in the United Kingdom, Elliott et al. reported a higher standardized mortality ratio for hypertension and cerebrovascular disease of borderline significance but no significant excess mortality from cerebrovascular disease in their geographical study [28].

In the exposed glucoproteinuria group, mortality risks for respiratory diseases increased in both genders. In the non-smokers, hazard risk ratios for respiratory diseases were also significantly increased in glucoproteinuria groups of both genders (Additional file 1: Table S3 and S4). Moreover, in the exposed glucoproteinuria group, deaths from acute bronchitis and pneumonia were 75% in men and 90% in women of deaths from respiratory diseases (60% in men and 70% in women in controls), suggesting increasing deaths from respiratory inflammation. Taken together, increased mortality for respiratory diseases may be caused from poor health status to get fatal inflammation due to renal dysfunction induced by Cd exposure, not due to smoking.

In women, mortality risks for digestive diseases for digestive diseases were higher in the exposed glucosuria group and glucoproteinuria group. In the glucoproteinuria group, the rate of deaths for gastrointestinal diseases was 65% of deaths for all digestive diseases, but 30% in the glucosuria group and 35% in the control no finding group. These results suggest that renal tubular dysfunction induced by Cd might increase body stress and increase gastrointestinal diseases such as peptic ulcer.

## Conclusion

Our findings indicate that the life prognosis of residents with renal dysfunction induced by Cd exposure was poor in the Jinzu River basin, Toyama, Japan, and diabetes, cardiovascular disease, particularly ischemic heart diseases, pneumonia and bronchitis, and renal diseases contributed to increasing mortality in both genders. Increased mortality risks for malignant neoplasms and digestive diseases were also evident in women with Cd nephropathy indicated by glucoproteinuria.

## Competing interest

All authors have approved the final version of the manuscript for publication, and declared all relevant competing interests. There is no competing interest for this paper.

## Authors’ contributions

HN, TK participated in the design and coordination of the study. KNa, MS, and MNa carried out preparation of survival data and creating data set. SM, YM, KNo, and RO carried out the survey for baseline data collection. MNi, SM, and YS performed the statistical analysis. SM, MNi, and HN prepared the draft for the manuscript. All authors read and approved the final manuscript.

## Supplementary Material

Additional file 1: Table S1 Levels of urinary Cd and positive rates of renal tubular markers at the second step of screening among four groups according to urinary findings at the first step in exposed subjects. **Table S2.** Codes of ICD 9 and 10 used in this study. **Table S3.** Hazard ratios for main causes of deaths in the groups with different urinary findings in male non-smokers without diabetes. **Table S4.** Hazard ratios for main causes of deaths in the groups with different urinary findings in female non-smokers without diabetes. Click here for file

Additional file 2: Table S1 Adjusted mortality risk ratios of inhabitants in the Cd polluted Jinzu River basin for 10 years of observations from total and specific causes of deaths as compared with controls. **Table S2.** Adjusted mortality risk ratios of inhabitants in the Cd polluted Jinzu River basin for 26 years of observations from total and specific causes of deaths as compared with controls. Click here for file
